# Learning robotic manipulation skills with multiple semantic goals by conservative curiosity-motivated exploration

**DOI:** 10.3389/fnbot.2023.1089270

**Published:** 2023-03-07

**Authors:** Changlin Han, Zhiyong Peng, Yadong Liu, Jingsheng Tang, Yang Yu, Zongtan Zhou

**Affiliations:** Department of Intelligence Science and Technology, College of Intelligence Science, National University of Defense Technology, Changsha, China

**Keywords:** hybrid policy mechanism, sparse reward, semantic goal, reinforcement learning, deep neural networks

## Abstract

Reinforcement learning (RL) empowers the agent to learn robotic manipulation skills autonomously. Compared with traditional single-goal RL, semantic-goal-conditioned RL expands the agent capacity to accomplish multiple semantic manipulation instructions. However, due to sparsely distributed semantic goals and sparse-reward agent-environment interactions, the hard exploration problem arises and impedes the agent training process. In traditional RL, curiosity-motivated exploration shows effectiveness in solving the hard exploration problem. However, in semantic-goal-conditioned RL, the performance of previous curiosity-motivated methods deteriorates, which we propose is because of their two defects: uncontrollability and distraction. To solve these defects, we propose a conservative curiosity-motivated method named mutual information motivation with hybrid policy mechanism (MIHM). MIHM mainly contributes two innovations: the decoupled-mutual-information-based intrinsic motivation, which prevents the agent from being motivated to explore dangerous states by uncontrollable curiosity; the precisely trained and automatically switched hybrid policy mechanism, which eliminates the distraction from the curiosity-motivated policy and achieves the optimal utilization of exploration and exploitation. Compared with four state-of-the-art curiosity-motivated methods in the sparse-reward robotic manipulation task with 35 valid semantic goals, including stacks of 2 or 3 objects and pyramids, our MIHM shows the fastest learning speed. Moreover, MIHM achieves the highest 0.9 total success rate, which is up to 0.6 in other methods. Throughout all the baseline methods, our MIHM is the only one that achieves to stack three objects.

## 1. Introduction

Enhanced by deep neural networks (DNNs), reinforcement learning (RL) (Sutton and Barto, 2018) empowers the agent to optimize its policy and solve difficult tasks by interacting with the task environment and exploiting the collected trajectories, which has made great breakthroughs in game playing (Vinyals et al., 2019), robotic locomotion (Hwangbo et al., 2019), robotic manipulation (Bai et al., 2019), etc. In standard RL, the policy is optimized for a single implicit goal embedded in the task, which cannot satisfy many practical tasks (e.g., robotic manipulation tasks) where the RL agent is required to understand multiple human control instructions and act toward various goals (Veeriah et al., 2018). Based on universal value function approximators (UVFAs) (Schaul et al., 2015), goal-conditioned RL (GCRL) (Colas et al., 2022) is proposed to accomplish these tasks by leveraging the goal-conditioned value network and policy network. The RL agent is optimized by goal-labeled trajectories with goal-specific rewards. However, when designing the goal-conditioned reward function, performing appropriate reward shaping (Badnava and Mozayani, 2019) for each goal is unrealistic, which makes the sparse reward setting become a common choice. Under this setting, the positive rewards are only sparsely set at some key nodes (e.g., when task goals are achieved). As a result of the lack of sufficient directive signals from the reward function, the RL agent inevitably meets the hard exploration problem (Ecoffet et al., 2019), which traps the policy optimization and goal attainment.

To overcome the hard exploration problem, because modifying goals in GCRL does not affect the environment dynamics, hindsight experience replay (HER) (Andrychowicz et al., 2017) is proposed to discover learning signals from the collected trajectories by relabeling the failed goal-reaching trajectories with their already achieved goals. However, this method only works fine when the task goals are continuous or densely distributed (e.g., setting the destination coordinates of objects as goals). For discrete or sparsely distributed goals in the form of semantic configurations (Akakzia et al., 2021) or natural language (Colas et al., 2020), which more conform to the human habits of giving instructions, the trajectories that can achieve the concerned goals account for a rather small proportion. Only from these trajectories can the goal relabeling method discover useful learning signals. The other trajectories cannot be finely evaluated and differentiated just by the sparse external rewards, no matter if goal relabeling is done.

**Figure 1:**
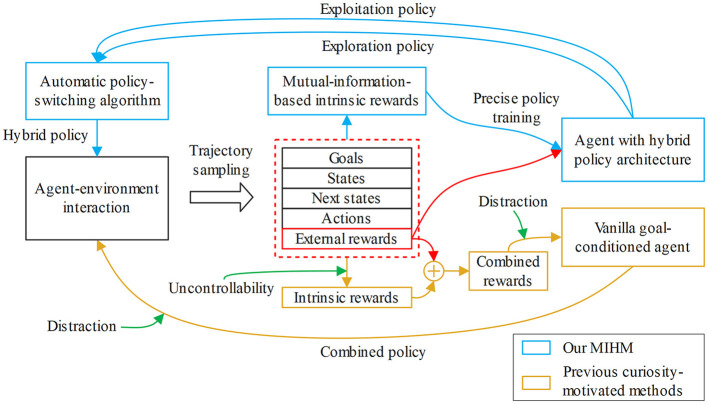
Comparison between our MIHM and previous curiosity-motivated methods. The previous curiosity-motivated methods have two defects, uncontrollability and distraction, which deteriorate their performance in semantic-GCRL. Comparatively, our MIHM contributes two innovations, the decoupled-mutual-information-based intrinsic rewards and hybrid policy mechanism, which effectively solve these defects.

In this paper, we focus on leveraging the sparse-reward GCRL to solve the robotic manipulation task with semantic goals. Since relying on only the external reward function makes it difficult to discover more useful learning signals, curiosity-motivated exploration methods become possible solutions, which generate intrinsic rewards to encourage the agent to explore novel states (Ostrovski et al., 2017; Burda et al., 2018b; Lee et al., 2020) or discover unlearned environment dynamics (Stadie et al., 2015; Houthooft et al., 2017; Pathak et al., 2017). However, the previous curiosity-motivated methods are not well compatible with the GCRL tasks, which we summarize into two aspects: uncontrollability and distraction. Because the agent cannot distinguish which novel states are more beneficial to the task, uncontrollability denotes that the task-irrelevant or even dangerous novelties will mislead the agent and cause the “noisy TV” problem (Pathak et al., 2017) to trap the exploration process. In curiosity-motivated methods, the agent policy is optimized by the weighted combination of the external rewards and the intrinsic rewards, which means the combined policy actually has two optimization objectives. Thus, the combined policy cannot be best optimized for the original goal-pursuing objective, and the agent will even be distracted by the dynamically varying intrinsic rewards to visit the intrinsic novelties instead of pursuing the goals. Comparison between our MIHM and previous curiosity-motivated methods is shown in Figure 1.

To accomplish the sparse-reward semantic-goal-conditioned robotic manipulation task by curiosity-motivated exploration, we propose a conservative curiosity-motivated exploration method named mutual information motivation with hybrid policy mechanism (MIHM), which successfully solves the defects of uncontrollability and distraction in the previous curiosity-motivated methods. The conservativeness in our method is embodied in two aspects. Firstly, we design a more conservative decoupled-mutual-information-based intrinsic reward generator, which encourages the agent to explore novel states with controllable behaviors. Secondly, the utilization of the curiosity-motivated exploration is more conservative. We construct a PopArt-normalized (Hessel et al., 2018) hybrid policy architecture, which detaches the goal-pursuing exploitation policy and precisely trains the curiosity-motivated exploration policy. Based on the two policies, we propose a value-function-based automatic policy-switching algorithm, which eliminates the distraction from the curiosity-motivated policy and achieves the optimal utilization of exploration and exploitation. In the robotic manipulation task proposed by Akakzia et al. (2021) with 35 different semantic goals, compared with the state-of-the-art curiosity-motivated methods, our MIHM shows the fastest learning speed and highest success rate. Moreover, our MIHM is the only one that achieves stacking three objects with just sparse external rewards.

## 2. Related work

Facing the hard exploration problem in sparse-reward semantic-GCRL, the agent is urgently required to improve its exploration ability toward unfamiliar states and unlearned semantically valid skills. An RL algorithm based on the DNNs can be more inclined to explore by adding action noise [e.g., the Gaussian noise or Ornstein-Uhlenbeck noise in deep deterministic policy gradients (Silver et al., n.d.)] or increasing action entropy [e.g., the entropy temperature adjustment in soft actor-critic (Haarnoja et al., 2018)]. However, lacking the exploitation of more environmental features, the above action-level exploration cannot help the agent to be aware of the states or state-action pairs that are potentially worth pursuing, which does not satisfy the circumstances when the state space or task horizon is expanded. Inspired by the intrinsic motivation mechanism in psychology (Oudeyer and Kaplan, 2008), intrinsically rewarding the novel state transitions is proven to be an effective method to motivate and guide the agent's exploration, which is named curiosity-motivated exploration. The intrinsic rewards are mainly generated for two purposes: increasing the diversity of the encountered states (Ostrovski et al., 2017; Burda et al., 2018b; Lee et al., 2020) and improving the agent's cognition of the environment dynamics (Stadie et al., 2015; Houthooft et al., 2017; Pathak et al., 2017).

For the first purpose, the intrinsic reward can be determined based on the pseudo count of the state (Ostrovski et al., 2017; Tang et al., 2017), where lower pseudo count means a rarer state and a higher reward. To gain adaptation to the high-dimensional and continuous state space, in recent years, the pseudo count has been realized by DNN-based state density estimation (Ostrovski et al., 2017) or hash-code-based state discretization (Tang et al., 2017). Moreover, the state novelty can also be calculated as the prediction error for a random distillation network (Burda et al., 2018b), which overcomes the inaccuracy of estimating the environment model. Another state novelty evaluation method is based on reachability (Savinov et al., 2018). By rewarding the states that cannot be reached from the familiar states within a certain number of steps, the intrinsic reward can be generated more directly and stably.

For the second purpose, the prediction error of the environment dynamics model can be used as the intrinsic reward. (Burda et al., 2018a) proved that, for training the environment dynamics model, it is necessary to use the encoded state space rather than the raw state space. They proposed an autoencoder-based state encoding function. (Pathak et al., 2017) proposed a self-supervised inverse dynamics model to learn to encode the state space, which is robust against the noisy TV problem. Moreover, the environment forward dynamics can be modeled by variational inference. (Houthooft et al., 2017) proposed motivating exploration by maximizing information gain about the agent's uncertainty of the environment dynamics by variational inference in Bayesian neural networks, which efficiently handles continuous state and action spaces.

In games (Vinyals et al., 2019) or robotic locomotion tasks (Hwangbo et al., 2019), the agent is often required to explore states as diverse as possible. The curiosity-based intrinsic rewards are consistent with the task objectives and show great performance. Moreover, replacing the traditional timestep-limited exploration rollouts, the infinite time horizon setting (Burda et al., 2018b) is often adopted in these tasks to further facilitate the discovery of novel information in the environment. However, in goal-conditioned robotic manipulation tasks, the agent is required to discover fine motor skills about the objects, which makes uncontrollably pursuing too diverse states easily cause interference. The intrinsic rewards are required to work as the auxiliaries for the external goal-conditioned rewards. Thus, it is necessary to improve the previous curiosity-motivated methods to solve the defects of uncontrollability and distraction. In our MIHM, we propose to improve the quality of intrinsic rewards and the utilization method of curiosity-motivated exploration.

## 3. Preliminaries

### 3.1. Goal-conditioned reinforcement learning

The multi-step policy-making problem that RL concerns can be formulated as a Markov decision process (MDP) (Sutton and Barto, 2018) M=<S, A, P, R, γ>, where S, A, P, R and γ represent the state space, action space, state transition probabilities, rewards, and discount factor, respectively. At timestep *t*, once interacting with the task environment, the agent can obtain a reward *r*_*t*_ for the state transition < *s*_*t*_, *a*_*t*_, *s*_*t*+1_ > by a predefined external reward function *r*. The discounted accumulation of future rewards is called return: $R_{t}=\overset{\infty }{\underset{i=t}{\sum }}\gamma^{i-t}r_{i}$. The policy π:S→A that RL optimizes is to maximize the expected return ${𝔼}_{s_{o}\sim p\left(s_{0}\right)}\left[V^{\pi }\left(s_{0}\right)\right]$, where the state value function $V^{\pi }\left(s_{t}\right)={𝔼}^{\pi }\left[R_{t}|s_{t}\right]$. In practice, instead of $V^{\pi }\left(s_{t}\right)$, the state-action value function $Q^{\pi }\left(s_{t},a_{t}\right)={𝔼}^{\pi }\left[R_{t}|s_{t},a_{t}\right]$ is often used, which can be updated by bootstrapping from the Bellman equation (Schaul et al., 2016). Leveraging the representation ability of the DNNs, the application scope of RL is extended from tabular cases to continuous state space or action space. The well-known RL algorithms include deep Q-networks (DQN) (Mnih et al., 2013), deep deterministic policy gradients (DDPG) (Silver et al., n.d.), twin delayed deep deterministic policy gradients (TD3) (Fujimoto et al., 2018), soft actor-critic (SAC) (Haarnoja et al., 2018).

In GCRL, the goal space G is additionally introduced, where each goal g∈G corresponds to an MDP $M_{g}=<S,\;A,\;P,\;R_{g},\;\gamma >$. Under different goals, the same transition will correspond to different rewards. To avoid the demand of the specific $V^{\pi_{g}}\left(s\right)$, $Q^{\pi_{g}}\left(s,a\right)$ and π_*g*_(*s*) for every goal *g*, UVFAs are proposed to use the DNN-based goal-conditioned *V*^π^(*s, g*), *Q*^π^(*s, a, g*) and π(*s, g*) to universally approximate all the $V^{\pi_{g}}\left(s\right)$, $Q^{\pi_{g}}\left(s,a\right)$ and π_*g*_(*s*). The optimization objective of GCRL becomes balancing all the goals and maximizing ${𝔼}_{\frac{s_{o}\sim p\left(s_{0}\right)}{g\sim p\left(g\right)}}\left[V^{\pi }\left(s_{0},g\right)\right]$. The universal approximators can be updated by the similar bootstrapping techniques in standard RL algorithms and are helpful to leverage the shared environmental dynamics across all the goals. Schaul et al. (2015) proved that, with the help of the generalizability of DNNs, the universal approximators can even generalize to the previously unseen goals, making it possible to use finite samples to learn policies for infinitely many or continuously distributed goals.

### 3.2. Semantic-goal-conditioned robotic manipulation

Compared with giving the precise destination coordinates, goals with semantic representations more conform to human habits and can contain more abstract and complicated intentions. In this paper, the semantic goal representations we concern are derived from Akakzia et al. (2021), where two semantic predicates, the *close* and the *on* binary predicates, *c*(·, ·) and *o*(·, ·), are defined to describe the spatial relations “close to” and “on the surface of” for the object pairs in the task environment. For example, *o*(*a, b*) = 1 expresses that object *a* is on the surface of object *b*. Furthermore, the joint activation of the predicates can express more complicated intentions. Because the *close* predicate has order invariance, considering the task with 3 objects *a*, *b* and *c*, a semantic goal *g* is the concatenation of 3 combinations of the *close* predicate and 6 permutations of the *on* predicate, as


(1) $$\begin{array}{l} g=[c\left(a,b\right),c\left(a,c\right),c\left(b,c\right),o\left(a,b\right),o\left(b,a\right),o\left(a,c\right),o\left(c,a\right),\\ \;o\left(b,c\right),o\left(c,b\right)]. \end{array}$$

**Figure 2:**
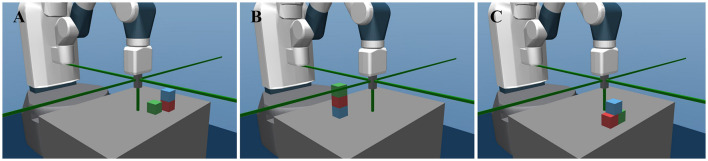
The robotic manipulation environment and three examples of the semantic configurations. **(A)** Shows the stack of 2 blocks, with the semantic configuration [111000100]. **(B)** Shows the stack of 3 blocks with the configuration [110011000]. **(C)** Shows a pyramid with the configuration [111000101].

Thus, in the semantic configuration space {0, 1}^9^, the agent can reach up to 35 physically valid goals, including stacks of 2 or 3 objects and pyramids, as Figure 2 shows. A simulation environment for this manipulation task is built based on the *MuJoCo* (Todorov et al., 2012) physics engine and *OpenAI Gym* interface (Brockman et al., 2016).

## 4. Methodology

### 4.1. Decoupled mutual information and intrinsic motivation

In the robotic manipulation task, instead of blindly pursuing state coverage or diversity, we think the exploratory behaviors toward the unfamiliar states must be more conservative and controllable. To model this controllable exploration paradigm, we adopt the information theoretic concept of mutual information. Particularly, we propose that the exploration objective is to maximize the mutual information *I* between the next state *S*′ and the current state-action pair *C*, where *C* is the concatenation of the current state *S* and action *A*. Using the definition of mutual information, *I* can be expressed as the differential of the entropy *H*:


(2) $$\begin{array}{l} I\left(S^{{}^{\prime }};C\right)=H\left(C\right)-H\left(C|S^{\prime }\right) \end{array}$$

(3) $$\begin{array}{l} =H\left(S^{\prime }\right)-H\left(S^{{}^{\prime }}|C\right). \end{array}$$

Equations 2, 3 are the inverse form and forward form of *I*(*S*′; *C*), respectively. Equation 2 means that to maximize *I*(*S*′; *C*), the agent is encouraged to increase the diversity of the state-action pairs [maximizing *H*(*C*)], while *C* is required to be unique when *S*′ is given [minimizing *H*(*C*|*S*′)]. Equation 3 means maximizing *I*(*S*′; *C*) corresponds to discovering more unfamiliar states [maximizing *H*(*S*′)], while *S*′ is predictable when the state-action pair is known [minimizing *H*(*S*′|*C*)]. Thus, *H*(*C*) or *H*(*S*′) represents the curiosity-based motivation while −*H*(*C*|*S*′) or −*H*(*S*′|*C*) represents the conservativeness. The mutual information *I*(*S*′; *C*) can be considered the KL-divergence between *p*(*s*′, *c*) and *p*(*s*′)*p*(*c*).


(4) $$\begin{array}{l} I\left(S^{{}^{\prime }};C\right)=D_{KL}\left(p\left(s^{{}^{\prime }},c\right)∥p\left(s^{\prime }\right)p\left(c\right)\right). \end{array}$$

Because the probability distributions of *s*′ and *c* are all unknown, following the mutual information neural estimator (MINE) (Belghazi et al., 2021), maximizing the KL-divergence can be represented as maximizing its Donsker-Varadhan lower bound. However, in practical RL tasks, because the initial ability of the agent is weak and it cannot initially acquire an extensive coverage of *s*′ and *c*, directly exploring to maximize the mutual information lower bound in the form of KL-divergence or JS-divergence (Kim et al., 2019) will make the agent more likely to confirm its actions in the experienced states than to explore the unfamiliar novel states (Campos et al., 2020). Consequently, the direct mutual-information-based exploration is too conservative to discover fine goal-conditioned manipulation skills with sparse rewards, while it is mainly adopted for unsupervised motion mode discovery (Eysenbach et al., 2018; Sharma et al., 2020) or high-operability state discovery (Mohamed and Rezende, 2015).

To explain this phenomenon, due to *p*(*s*′, *c*) = *p*(*s*′|*c*)*p*(*c*), we rewrite $D_{KL}\left(p\left(s^{{}^{\prime }},c\right)∥p\left(s^{{}^{\prime }}\right)p\left(c\right)\right)$ as


(5) $$\begin{array}{l} D_{KL}\left(p\left(s^{{}^{\prime }},c\right)∥p\left(s^{{}^{\prime }}\right)p\left(c\right)\right)=\int p\left(s^{{}^{\prime }},c\right)\log\frac{p\left(s^{{}^{\prime }}|c\right)}{p\left(s^{{}^{\prime }}\right)}ds^{\prime }dc\\ \;={𝔼}_{p\left(s^{{}^{\prime }},c\right)}\left[\log\frac{p\left(s^{{}^{\prime }}|c\right)}{p\left(s^{{}^{\prime }}\right)}\right] \end{array}$$

where *s*′, *c* are sampled from the RL rollouts with the agent's current policy π. The mutual information *I*(*S*′; *C*) can be maximized by optimizing the agent's policy in an RL manner with the intrinsic reward function $r_{int}=\log q\left(s^{{}^{\prime }}|c\right)-\log q\left(s^{\prime }\right)$, where *q*(*s*′|*c*) and *q*(*s*′) are the online estimations of *p*(*s*′|*c*) and *p*(*s*′) based on the collected < *s*′, *c*>. Assuming that *q*(*s*′) can be approximated by plenty of *q*(*s*′|*c*), i.e., $q\left(s^{{}^{\prime }}\right)=\frac{1}{N}\underset{\forall c_{i}}{\sum }q\left(s^{{}^{\prime }}|c_{i}\right)$, the intrinsic reward can be rewritten as


(6) $$\begin{array}{l} r_{int}=\log q\left(s^{{}^{\prime }}|c\right)-\log\frac{1}{N}\underset{\forall c_{i}}{\sum }q\left(s^{{}^{\prime }}|c_{i}\right)\\ \;=\log\frac{q\left(s^{{}^{\prime }}|c\right)}{\underset{\forall c_{i}}{\sum }q\left(s^{{}^{\prime }}|c_{i}\right)}+\log N\\ \;=\log\frac{q\left(s^{{}^{\prime }}|c\right)}{1+\sum_{\forall c_{i}\neq c}\frac{q(s^{{}^{\prime }}|c_{i})+\epsilon }{q(s^{{}^{\prime }}|c)+\epsilon }}+\log N. \end{array}$$

In the experienced states, for *s*′ generated from *c*, the forward dynamics *q*(*s*′|*c*) is updated to be close to 1. For other *c*_*i*_ ≠ *c*, $q\left(s^{{}^{\prime }}|c_{i}\right)$ is close to 0. Therefore, the typical intrinsic reward *r*_*int*_ ≈ *log*1 + log*N* = *logN* > 0. Comparatively, in the unexperienced states, for any *c*_*i*_, $q\left(s^{{}^{\prime }}|c_{i}\right)$ is nearly 0. The typical intrinsic reward $r_{int}^{{}^{\prime }}\approx \log\left(\frac{1}{N}\right)+\log N=0<r_{int}$. Thus, the agent is more likely to obtain higher intrinsic rewards in the experienced states, which prevents its exploration to the unfamiliar states.

To solve this problem, different from (Kim et al., 2019; Belghazi et al., 2021), we propose to decouple the calculation of mutual information and respectively maximize the two entropy components *H*(*S*′) and −*H*(*S*′|*C*) in Equation 3 with different paces. The pace of *H*(*S*′) is fixed and the pace of −*H*(*S*′|*C*) is adjusted with a decay factor to ensure a curiosity-motivated, conservativeness-corrected exploration. We firstly introduce how to maximize *H*(*S*′) and −*H*(*S*′|*C*) then the adjustment of the pace. To approximate $H\left(S^{\prime }\right)=-{𝔼}_{p\left(s^{\prime }\right)}\log\left[p\left(s^{\prime }\right)\right]$, because *p*(*s*′) is high-dimensional and hard to be estimated, we adopt the non-parametric particle-based entropy estimator proposed by Singh et al. (2003) that has been widely researched in statistics (Jiao et al., 2018). Considering a sampled dataset ${\left\{{s^{\prime }}_{i}\right\}}_{i=1}^{N}$, *H*(*S*′) can be approximated by considering the distance between each ${s^{\prime }}_{i}$ and its *k*th nearest neighbor.


(7) $$\begin{array}{l} {Ĥ}_{particle}\left(S^{\prime }\right)=\frac{1}{N}\overset{N}{\underset{i=1}{\sum }}\log\frac{N\cdot ||{s^{{}^{\prime }}}_{i}-{s^{{}^{\prime }}}_{i}^{k-NN}|{|}_{2}^{D_{S^{{}^{\prime }}}}\cdot \pi^{\frac{D_{S^{{}^{\prime }}}}{2}}}{k\cdot \Gamma \left(\frac{D_{S^{{}^{\prime }}}}{2}+1\right)}+b\left(k\right) \end{array}$$

(8) $$\begin{array}{l} \propto \frac{1}{N}\overset{N}{\underset{i=1}{\sum }}\log\|{s^{{}^{\prime }}}_{i}-{s^{{}^{\prime }}}_{i}^{k-NN}{\|}_{2} \end{array}$$

where ${s^{\prime }}_{i}^{k-NN}$ denotes the *k*th nearest neighbor of ${s^{\prime }}_{i}$ in the dataset ${\left\{{s^{\prime }}_{i}\right\}}_{i=1}^{N}$, *b*(*k*) denotes a bias correction term that only depends on the hyperparameter *k*, $D_{S^{{}^{\prime }}}$ is the dimension of *s*′, Γ is the gamma function, and ||·||_2_ denotes the Euclidean distance. The transition from Equations 7, 8 always holds for $D_{S^{{}^{\prime }}}>0$. To maximize *H*(*S*′), we can treat each sampled transition < *s*′, *c*> as a particle (Seo et al., 2021). Following (Liu and Abbeel, 2021), we use the average distance over all *k* nearest neighbors for a more robust approximation, so the intrinsic reward $r_{int}^{H\left(S^{{}^{\prime }}\right)}$ is designed as


(9) $$\begin{array}{l} r_{int}^{H\left(S^{{}^{\prime }}\right)}=\log\left(m+\frac{1}{k}\underset{{s^{i}}_{i}^{k-NN}\in N_{k}\left({s^{{}^{\prime }}}_{i}\right)}{\sum }\|{s^{{}^{\prime }}}_{i}-{s^{{}^{\prime }}}_{i}^{k-NN}{\|}_{2}\right) \end{array}$$

where *m* = 1 is a constant for numerical stability, $N_{k}\left({s^{{}^{\prime }}}_{i}\right)$ denotes the set of *k* nearest neighbors around ${s^{{}^{\prime }}}_{i}$.

Compared with *p*(*s*′), the posterior probability *p*(*s*′|*c*) in $-H\left(S^{{}^{\prime }}|C\right)={𝔼}_{p\left(s^{\prime },c\right)}\log\left[p\left(s^{\prime }|c\right)\right]$ is relatively easier to be estimated, because it follows the forward dynamics and can be simply treated as a Gaussian distribution. Thus, we leverage a factored Gaussian DNN $D_{G}\left(s^{\prime }|c;\psi \right)$ with the reparameterization trick (Li et al., 2017) to predict *p*(*s*′|*c*), which is updated by descending with gradients $-{𝔼}_{p\left(s^{\prime },c\right)}\left[\nabla_{\psi }\log D_{G}\left(s^{\prime }|c;\psi \right)\right]$. Actually, ${𝔼}_{p\left(s^{\prime },c\right)}\log\left[D_{G}\left(s^{\prime }|c\right)\right]$ is the lower bound of −*H*(*S*′|*C*) and becomes tight when 𝔼_*p*(*c*)_[*D*_*KL*_(*p*(·|*c*)||*D*_*G*_(·|*c*))] → 0 (Chen et al., 2016). We use $D_{G}\left(s^{\prime }|c\right)$ to intrinsically reward each sampled transition < *s*′, *c*>. Thus, to maximize −*H*(*S*′|*C*), the intrinsic reward $r_{int}^{H\left(S^{{}^{\prime }}|C\right)}$ is designed as


(10) $$\begin{array}{l} r_{int}^{-H\left(S^{{}^{\prime }}|C\right)}=\log\left[m+D_{G}\left(s^{\prime }|c\right)\right] \end{array}$$

where *m* = 1 is a constant for numerical stability.

Based on Equations 9, 10, considering the adjusting pace λ for −*H*(*S*′|*C*) to control the conservativeness, the whole intrinsic reward is represented as


(11) $$\begin{array}{l} \;r_{int}=r_{int}^{H\left(S^{{}^{\prime }}\right)}+\lambda \cdot r_{int}^{-H\left(S^{{}^{\prime }}|C\right)}\\ \lambda =\left[\min\left(1-\xi^{ep},\;\beta \right)\right]\cdot \frac{\sigma_{S^{{}^{\prime }}}}{\sigma_{S^{{}^{\prime }}|C}} \end{array}$$

where 0 < ξ < 1 is the decaying factor, *ep* is the number of training epoch, β < 1 is the cutoff threshold for the increasing 1 − ξ^*ep*^, $\sigma_{S^{{}^{\prime }}}$ and $\sigma_{S^{{}^{\prime }}|C}$ are the running estimated standard deviations of previously generated $r_{int}^{H\left(S^{{}^{\prime }}\right)}$ and $r_{int}^{-H\left(S^{{}^{\prime }}|C\right)}$. The adjusting pace λ serves two purposes: *min*(1 − ξ^*ep*^, β) controls the proportion of the conservativeness part $r_{int}^{-H\left(S^{{}^{\prime }}|C\right)}$ especially in the early stage of the training process to encourage the curiosity-based exploration; $\frac{\sigma_{S^{{}^{\prime }}}}{\sigma_{S^{{}^{\prime }}|C}}$ balances the variation amplitude of $r_{int}^{H\left(S^{{}^{\prime }}\right)}$ and $r_{int}^{-H\left(S^{{}^{\prime }}|C\right)}$ for better proportionality of the curiosity-based part and the conservativeness part. The decoupled-mutual-information-based intrinsic reward is actually a conservative curiosity-motivated intrinsic reward, which encourages the agent to explore diverse states but penalizes the uncontrollable actions or states.

### 4.2. Hybrid policy architecture with PopArt normalization

Traditionally, in the curiosity-motivated goal-conditioned robotic manipulation task, the agent policy is a combined policy π_*c*_, and the reward of each experienced transition is the weighted sum of the external reward and the z-score normalized intrinsic reward: *r*_*c*_ = *r*_*ext*_+τ·*n*_*r*_(*r*_*int*_), where τ is the proportionality coefficient, and *n*_*r*_(·) represents the reward normalization that is necessary in proportionating the dynamically varying *r*_*int*_. On the one hand, the intrinsic reward *r*_*int*_ facilitates exploration and assists the agent in discovering more external rewards. On the other hand, the existence of the varying *r*_*int*_ interferes with the original optimization of the goal-pursuing policy and will even cause the agent to visit the intrinsic novelties but not to pursue the task goals. Thus, we think it is necessary to construct a hybrid policy architecture to detach the goal-pursuing exploitation policy π_*d*_ from the curiosity-motivated combined exploration policy π_*c*_. Then, by automatically switching between the two policies, a better hybrid policy π_*hybrid*_ can be obtained and adopted in the trajectory sampling of the RL training process (introduced in value-function-based policy-switching algorithm section), which eliminates the distraction from curiosity-motivated policy π_*c*_. The hybrid policy architecture and the policy-switching algorithm constitute our hybrid policy mechanism.

Note that the hybrid policy architecture must be updated by the off-policy RL algorithms, because a shared experience buffer B is leveraged in the updates, where the stored trajectories are sampled by the hybrid policy π_*hybrid*_. A straightforward hybrid policy architecture can be constructed by using the combined reward *r*_*c*_ = *r*_*ext*_+τ·*n*_*r*_(*r*_*int*_) to train π_*c*_ and using *r*_*d*_ = *r*_*ext*_ to train π_*d*_. However, because the dynamic *r*_*int*_ has varying mean and variance, the output precision of the combined exploration Q-function *Q*_*c*_(*s*_*t*_, *a*_*t*_, *g*) will be decreased once the reward normalizer *n*_*r*_(·) is updated (van Hasselt et al., 2016). Moreover, a combined reward function is adverse to making the utmost of every reward component (van Seijen et al., 2017). Thus, it is necessary to propose a better way to train *Q*_*c*_(*s*_*t*_, *a*_*t*_, *g* ).

For the combined reward *r*_*c*_ and the shared trajectory-sampling policy, there exists


(12) $$\begin{array}{l} Q_{c}\left(s_{t},a_{t},g\right)=𝔼\left[\overset{\infty }{\underset{t=0}{\sum }}\gamma^{t}r_{c}|s_{t},a_{t},g,s_{t+1}\;\right]\\ \;=𝔼\left[\overset{\infty }{\underset{t=0}{\sum }}\gamma^{t}\left(r_{ext}+\tau \cdot n_{r}\left(r_{int}\right)\right)|s_{t},a_{t},g,s_{t+\;1}\right]\\ \;=𝔼\left[\overset{\infty }{\underset{t=0}{\sum }}\gamma^{t}r_{ext}|s_{t},a_{t},g,s_{t+1}\right]\\ \;+\tau \cdot 𝔼\left[\overset{\infty }{\underset{t=0}{\sum }}\gamma^{t}n_{r}\left(r_{int}\right)|s_{t},a_{t},s_{t+1}\;\right]\\ \;=Q_{ext}\left(s_{t},a_{t},g\right)+\tau \cdot Q_{int}^{n_{r}}\left(s_{t},a_{t}\right). \end{array}$$

According to Equation 12, for the optimization of π_*c*_, learning the Q-function *Q*_*c*_(*s*_*t*_, *a*_*t*_, *g*) with the combined reward *r*_*c*_ is equal to learning and combining the external Q-function *Q*_*ext*_(*s*_*t*_, *a*_*t*_, *g*) and the reward-normalized intrinsic Q-function $Q_{int}^{n_{r}}\left(s_{t},a_{t}\right)$. Here, we adopt the PopArt normalization for the Q-network (Hessel et al., 2018), *n*_*PopArt*_(*Q*_*int*_(*s*_*t*_, *a*_*t*_)), to replace the reward-normalized $Q_{int}^{n_{r}}\left(s_{t},a_{t}\right)$, which not only adaptively normalizes the Q-values to fluctuate around 0 (similar to $Q_{int}^{n_{r}}\left(s_{t},a_{t}\right)$) without breaking the original reward function structure (Schulman et al., 2018), but also preserves the output precision of the Q-network against the varying mean and variance of the normalizer. Thus, the combined Q-function is *Q*_*c*_(*s*_*t*_, *a*_*t*_, *g*) = *Q*_*ext*_(*s*_*t*_, *a*_*t*_, *g*) + τ · *n*_*PopArt*_(*Q*_*int*_(*s*_*t*_, *a*_*t*_ )).

**Figure 3:**
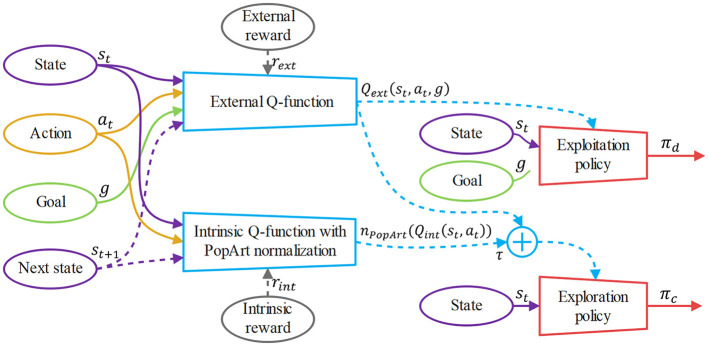
The overview of our hybrid policy architecture. The solid arrows show the inputs and outputs of the Q-functions and policies, while the dotted arrows show the additional sources used for the updates of the Q-functions and policies. The external Q-function and the intrinsic Q-function are updated by the Bellman bootstrapping with *r*_*ext*_ and *r*_*int*_, respectively. After the intrinsic Q-function is PopArt-normalized, the exploitation policy π_*d*_ is updated by the gradient ascent of *Q*_*ext*_(*s*_*t*_, *a*_*t*_, *g*) and the exploration policy π_*c*_ is updated by the gradient ascent of *Q*_*ext*_(*s*_*t*_, *a*_*t*_, *g*)+τ·*n*_*PopArt*_(*Q*_*int*_(*s*_*t*_, *a*_*t*_)).

Our hybrid policy architecture is shown in Figure 3. The combined exploration policy π_*c*_ is optimized by minimize the KL-divergence between *Q*_*c*_(*s*_*t*_, *a*_*t*_, *g*) and π_*c*_:


(13) $$\begin{array}{l} J_{\pi_{c}}\left(\theta^{\pi_{c}}\right)={𝔼}_{s_{i}\sim D}[D_{KL}(\pi_{c}\left(\cdot |s_{i},g;\theta^{\pi_{c}}\right)||\\ \frac{\exp\left(\frac{1}{\alpha }\left(Q_{ext}\left(s_{i},\cdot ,g\right)+\tau \cdot n_{PopArt}\left(Q_{int}\left(s_{i},\cdot \right)\right)\right)\right)}{Z_{c}\left(s_{i}\right)})]. \end{array}$$

where $Z_{c}\left(s_{i}\right)=\underset{a_{i}}{\sum }\exp\left(\frac{1}{\alpha }\left(Q_{ext}\left(s_{i},\cdot ,g\right)+\tau \cdot n_{PopArt}\left(Q_{int}\left(s_{i},\cdot \right)\right)\right)\right)$ is the normalization constant and can be omitted in the optimization.

Similarly, the exploitation policy π_*d*_ is optimized by minimize the KL-divergence between *Q*_*d*_(*s*_*t*_, *a*_*t*_, *g*) = *Q*_*ext*_(*s*_*t*_, *a*_*t*_, *g*) and π_*d*_:


(14) $$\begin{array}{l} J_{\pi_{d}}\left(\theta^{\pi_{d}}\right)={𝔼}_{s_{i}\sim D}\left[D_{KL}\left(\pi_{d}\left(\cdot |s_{i},g;\theta^{\pi_{d}}\right)||\frac{\exp\left(\frac{1}{\alpha }Q_{ext}\left(s_{i},\cdot ,g\right)\right)}{Z_{d}\left(s_{i}\right)}\right)\right]. \end{array}$$

where $Z_{d}\left(s_{i}\right)=\underset{a_{i}}{\sum }\exp\left(\frac{1}{\alpha }Q_{ext}\left(s_{i},\cdot ,g\right)\right)$ is the normalization constant.

### 4.3. Value-function-based policy-switching algorithm

As introduced in hybrid policy architecture with PopArt normalization section, the combined Q-function *Q*_*c*_(*s*_*t*_, *a*_*t*_, *g*) is constituted by two parts, where the curiosity-based part is normalized and dynamically varies around 0. However, pursuing semantic goals (especially complicated semantic goals) cannot avoid leveraging learned skills or trajectories with negative novelty. Thus, in the previous curiosity-motivated methods that only adopt the combined policy, the distraction occurs when pursuing goals following part of the familiar trajectories has less attraction than visiting the novelties, i.e., ∃s∈S,  ∃g∈G, *Q*_*c*_(*s, a*_*curiosity*_, *g*) > *Q*_*c*_(*s, a*_*goal*_, *g*), where *a*_*curiosity*_ denotes the action toward the novelties and *a*_*goal*_ denotes the action toward the goals. Based on the hybrid policy architecture, our detached exploitation policy π_*d*_ is unaffected by the intrinsic rewards, whose Q-function can reflect the more accurate expected return of goal pursuing. Thus, we propose the following hybrid policy π_*hybrid*_ switching between π_*d*_ and π_*c*_ for every (*s, g*) and prove that it takes advantage of both π_*d*_ and π_*c*_.


(15) $$\begin{array}{l} \pi_{hybrid}\left(s,g\right)=\{\begin{array}{c} \pi_{d}\left(s,g\right)\;V_{c}\left(s,g\right)<V_{d}\left(s,g\right)\\ \pi_{c}\left(s,g\right)\;V_{c}\left(s,g\right)\geq V_{d}\left(s,g\right) \end{array} \end{array}$$

where *V*_*c*_(*s, g*) = 𝔼_*a*_*c*_~ π_*c*_(*s, g*)_*Q*_*c*_(*s, a*_*c*_, *g*), *V*_*d*_(*s, g*) = 𝔼_*a*_*d*_~ π_*d*_(*s, g*)_*Q*_*d*_(*s, a*_*d*_, *g*). In the algorithm implementation, for simplicity, we do not train additional V-networks and use Q_c_(s, a_c_, g), Q_d_(s, a_d_, g) to approximate *V*_*c*_(*s, g*) and *V*_*d*_(*s, g*). Assuming there exists a *V*_*hybrid*_(*s, g*) for policy π_*hybrid*_, we prove ∀s∈S,  ∀g∈G, *V*_*hybrid*_(*s, g*) ≥ *V*_*c*_(*s, g*), *V*_*hybrid*_(*s, g*) ≥ *V*_*d*_(*s, g* ).

At a state *s*_*i*_ ∈ *S*, g∈G, we define the advantageous policy between π_*d*_ and π_*c*_ as


(16) $$\begin{array}{l} \pi_{adv}^{s_{i},g}\left(s,g\right)=\{\begin{array}{c} \pi_{d}\left(s,g\right)\&V_{c}\left(s_{i},g\right)<V_{d}\left(s_{i},g\right)\\ \pi_{c}\left(s,g\right)\&V_{c}\left(s_{i},g\right)\geq V_{d}\left(s_{i},g\right) \end{array}. \end{array}$$

Obviously, we have $V_{adv}^{s_{i},g}\left(s_{i},g\right)\geq V_{d}\left(s_{i},g\right)$, $V_{adv}^{s_{i},g}\left(s_{i},g\right)\geq V_{c}\left(s_{i},g\right)$ and $V_{adv}^{s_{i},g}\left(s_{i},g\right)\geq V_{adv}^{s^{{}^{\prime }},g}\left(s_{i},g\right)$, where *s*′ is another state different from *s*_*i*_. Compared with the hybrid policy π_*hybrid*_ in Equation 15, $\pi_{adv}^{s_{i},g}$ can be considered as switching between π_*d*_ and π_*c*_ only once at (*s*_*i*_, *g*). Starting from state *s*_*i*_, we follow policy π_*hybrid*_ for *n* steps and then follow $\pi_{adv}^{s_{i+n},g}$. A value function is obtained as


(17) $$\begin{array}{l} V_{n}\left(s_{i},g\right)=\{\begin{array}{c} {𝔼}_{\left(s_{i+1},r_{i}\right)\sim \pi_{hybrid}\left(s_{i},g\right)}\left[r_{i}+\gamma V_{n-1}\left(s_{i+1},g\right)\right]\&n\geq 1\\ V_{adv}^{s_{i},g}\left(s_{i},g\right)\&n=0 \end{array}. \end{array}$$

When *n* = 1, there exists


(18) $$\begin{array}{l} V_{1}\left(s_{i},g\right)={𝔼}_{\left(s_{i+1},r_{i}\right)\sim \pi_{hybrid}\left(s_{i},g\right)}\left[r_{i}+\gamma V_{0}\left(s_{i+1},g\;\right)\right]\\ \;={𝔼}_{\left(s_{i+1},r_{i}\right)\sim \pi_{hybrid}\left(s_{i},g\right)}\left[r_{i}+\gamma V_{adv}^{s_{i+1},g}\left(s_{i+1},g\right)\;\right]\\ \;\geq {𝔼}_{\left(s_{i}+1,r_{i}\right)\sim \pi_{hybrid}\left(s_{i},g\right)}\left[r_{i}+\gamma V_{adv}^{s_{i},g}\left(s_{i+1},g\;\right)\right]\\ \;={𝔼}_{\left(s_{i}+1,r_{i}\right)\sim \pi_{adv}^{s_{i},g}\left(s_{i},g\right)}\left[r_{i}+\gamma V_{adv}^{s_{i},g}\left(s_{i+1},g\;\right)\right]\\ \;=V_{adv}^{s_{i},g}\left(s_{i},g\;\right)\\ \;=V_{0}\left(s_{i},g\right). \end{array}$$

By induction, we obtain ∀*n* ≥ 1, $V_{n}\left(s_{i},g\right)\geq V_{n-1}\left(s_{i},g\right)\geq \cdots \geq V_{0}\left(s_{i},g\right)=V_{adv}^{s_{i},g}\left(s_{i},g\right)\geq V_{c}\left(s_{i},g\right)$ and *V*_*n*_(*s*_*i*_, *g*) ≥ *V*_*d*_(*s*_*i*_, *g*). When *n* → ∞, we have *V*_*hybrid*_(*s, g*) ≥ *V*_*c*_(*s, g*) and *V*_*hybrid*_(*s, g*) ≥ *V*_*d*_(*s, g*). In our task, because of the fluctuations of the curiosity-based part of the combined exploration policy, at some states *V*_*c*_(*s, g*) > *V*_*d*_(*s, g*) and at other states *V*_*d*_(*s, g*) > *V*_*c*_(*s, g*). On this occasion, *V*_*hybrid*_(*s, g*) > *V*_*c*_(*s, g*) and *V*_*hybrid*_(*s, g*) > c*V*_*d*_(*s, g*), which means that π_*hybrid*_ is strictly better than π_*d*_ and π_*c*_. Thus, our π_*hybrid*_ can automatically switch between goal-pursuing and novelty-visiting, reducing the distraction from curiosity-based motivation as much as possible.

Note that we only implement the policy-switching algorithm in the RL training process. In the RL evaluation process, because curiosity-motivated exploration is unnecessary, we adopt only the exploitation policy π_*d*_. In conclusion, the whole pseudocode of our MIHM is available in Algorithm 1.

**Algorithm 1:** Mutual information motivation with hybrid policy mechanism (MIHM).

**Require:** Q-function $Q_{ext}^{\pi }\left(s_{t},a_{t},g\right)$ and $Q_{int}^{\pi }\left(s_{t},a_{t}\right)$, policy π_*d*_ and π_*c*_, a factored Gaussian network $D_{G}\left(s^{\prime }|c;\psi \right)$, a replay buffer B, a semantic goal set G
1: Initialize *Q*_*ext*_(*s*_*t*_, *a*_*t*_, *g*), *Q*_*int*_(*s*_*t*_, *a*_*t*_), π_*d*_, π_*c*_, $D_{G}\left(s^{\prime }|c;\psi \right)$, B
2: **for** *epoch* = 1 *to L* **do**
3: **for** *rollout* = 1 *to M* **do**
4: Initialize the task environment and the desired goal *g*
5: **for** *timestep* = 1 *to T* **do**
6: Interact with the environment by π_*hybrid*_ toward *g* by Equation 15
7: **end for**
8: Store the transitions of the rollout in B
9: **for** *step* = 1 *to N* **do**
10: Sample minibatch *B* from B and do goal relabeling by HER
11: Calculate $r_{int}^{H\left(S^{{}^{\prime }}\right)}$, $r_{int}^{-H\left(S^{{}^{\prime }}|C\right)}$, λ and intrinsic rewards *r*_*int*_ by Equations 9–11
12: Update *Q*_*ext*_(*s*_*t*_, *a*_*t*_, *g*), *n*_*PopArt*_(*Q*_*int*_(*s*_*t*_, *a*_*t*_))
13: Update π_*c*_, π_*d*_ by Eqs 13, 14
14: **end for**
15: **end for**
16: **end for**

## 5. Experiments

### 5.1. Experiment settings

As introduced in semantic-goal-conditioned robotic manipulation section, we adopt the semantic-goal-conditioned robotic manipulation task derived from Akakzia et al. (2021) for experiments. In the task, the actions of the agent are 4-dimensional: 3 dimensions for the gripper velocities and 1 dimension for the grasping velocity. The state observation is 55-dimensional: the agent can observe the Cartesian and angular positions and velocities of its gripper and the objects. The currently achieved goal *g*_*ac*_ is available for the agent. A binary sparse reward setting is adopted as


(19) $$\begin{array}{l} r_{g}\left(s,a,s^{\prime }\right)≜\{\begin{array}{l} 1,\;\\ \;\phi \left(s^{{}^{\prime }}\right)=g\\ 0,\;\\ \;otherwise \end{array} \end{array}$$

where ϕ(s): S→G is the function to abstract the achieved goal *g*_*ac*_ from state *s*.

In our experiments, we adopt four state-of-the-art algorithms to compare with our MIHM, including intrinsic curiosity module (ICM) (Pathak et al., 2017) and random network distillation (RND) (Burda et al., 2018b), diversity actor-critic (DAC) (Han and Sung, 2021), random encoders for efficient exploration (RE3) (Seo et al., 2021). The UVFA-based off-policy RL algorithm soft actor-critic (SAC) (Haarnoja et al., 2018) is adopted for the agent, where the goal-conditioned Q-networks and policy networks are constructed by the Deep Sets (Zaheer et al., 2018). When implementing each algorithm, we use 500 epochs with 16 CPU workers running on 16 different initialization seeds and the policy evaluation is based on the average performance over the 16 seeds. Each epoch has 50 cycles while each cycle has 2 rollouts. To avoid interference from the task-irrelevant states, different from the previous curiosity-motivated methods, we do not adopt the infinite time horizon setting. Instead, each rollout has a fixed horizon of 50 timesteps. We set *k* in Equation 9 for the *k*-NN-based particle entropy estimator as 3, β and γ in Equation 11 as 0.7 and 0.99, the policy combination proportionality coefficient τ in Equation 13 as 0.2. To facilitate the training process, we adopt a biased initialization trick (Akakzia et al., 2021): after 80 epochs, the task environment is initialized with stacks of 2 blocks 21% of times, stacks of 3 blocks 9% of times, and a block is initially put in the agent's gripper 50% of times. We also utilize a simple curriculum learning setting: the desired goals of the rollouts are uniformly sampled in the already visited semantic goals, which means the agent will not be assigned goals that are too hard at the early stage of training.

### 5.2. Results and analyses

To facilitate the presentation and comparison of results, according to the number of layers the objects are desired to be stacked into, we classify the semantic goals into three categories: one-layer goals, two-layer goals and three-layer goals. Achieving the one-layer goals only requires the agent to realize the *close* predicates. Achieving the two-layer goals requires the agent to discover the stack skill and realize the *on* predicates. Achieving the three-layer goals requires the sophisticated stacking skill. The number of goals belonging to each category is shown in Table 1.

**Table 1:** The number of goals in each goal category.

**Categories**	**One-layer goals**	**Two-layer goals**	**Three-layer goals**	**Total**
Number of goals	8	21	6	35

**Figure 4:**
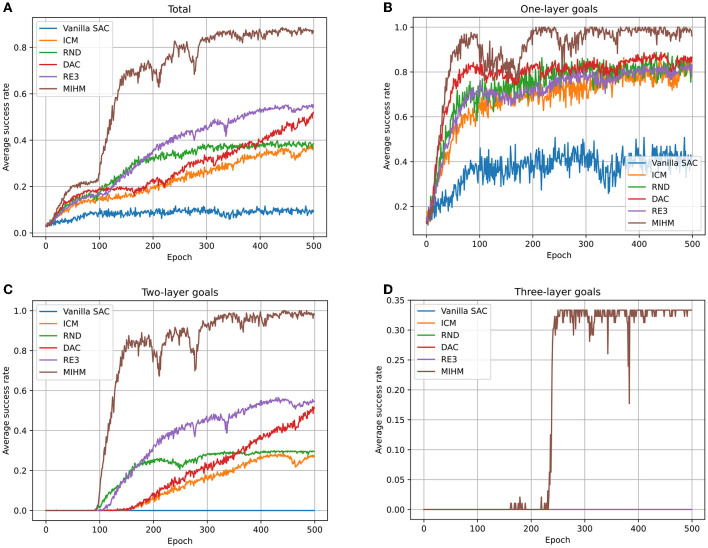
Learning processes of six algorithms (vanilla SAC, ICM, RND, DAC, RE3 and MIHM) for different categories of goals. **(A)** Shows the variations of average success rates of all 35 goals. **(B–D)** Show the variations of the average success rates of one-layer goals, two-layer goals and three-layer goals, respectively. Vanilla SAC agent can only achieve some of the one-layer goals with low success rates. ICM, RND, DAC, and RE3 enable the agent to achieve most of the one-layer goals and some of the two-layer goals. Comparatively, our MIHM enables the agent to learn all one-layer goals and two-layer goals. For the three-layer goals, our MIHM obtains an average success rate of 33%.

We record the learning processes of six algorithms (vanilla SAC, ICM, RND, DAC, RE3 and MIHM) in Figure 4. The number of learned semantic goals (whose success rates are >80%) for each category is shown in Table 2. It is shown that the sparse-reward semantic-goal-conditioned robotic manipulation is a rather difficult task for the vanilla SAC. Without curiosity-motivated exploration, only by random exploration cannot the agent obtain sufficient learning signals. After 500 epochs, the vanilla SAC agent cannot fully learn the one-layer goals. Comparatively, the curiosity-motivated methods effectively improve the agent performance, which make it possible to achieve some of the two-layer goals after epoch 80 (because our biased initialization trick starts to work in epoch 80). However, none of the success rates of two-layer goals in RND and ICM can be stabilized above 80%. RND performs slightly better than ICM, because by leveraging the random target-encoding network, RND overcomes the problem in ICM that the agent cannot distinguish the novelty of state-action pairs from the randomness of the environmental forward dynamics. DAC and RE3 improve the efficiency perform better than RND and ICM, achieving some of the two-layer goals. However, due to the two defects of curiosity-motivated methods, the four baseline methods cannot achieve the three-layer goals. Our MIHM solves these defects and shows the best performance, learning up to 31 goals and is the only one to achieve three-layer goals.

**Table 2:** The number of finally learned goals (whose success rates are >80%) in each goal category for four algorithms.

**Algorithms**	**One-layer goals**	**Two-layer goals**	**Three-layer goals**	**Total**
Vanilla SAC	1	0	0	1
ICM	4	0	0	4
RND	4	0	0	4
DAC	6	3	0	9
RE3	7	5	0	12
MIHM	8	21	2	31

**Figure 5:**
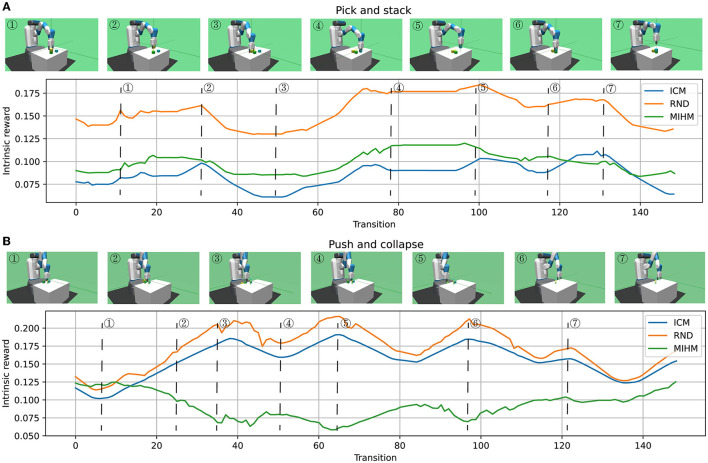
The variations of intrinsic rewards when picking and stacking objects **(A)** and pushing objects off the table **(B)**. **(A)** Shows that ICM, RND and MIHM can effectively reward the novel and controllable behaviors. **(B)** Shows that ICM and RND wrongly reward the novel but uncontrollable behaviors. Comparatively, our MIHM can effectively discover and prevent the uncontrollable behaviors.

To further illustrate the differences among the intrinsic rewards generated by MIHM and other curiosity-motivated methods, we take ICM and RND as comparisons and artificially control the robotic arm for two episodes: one episode is to pick and stack objects; the other is to push objects off the table. These two episodes reflect the typical scenarios that are novel and controllable, novel but uncontrollable. We store the intrinsic reward generators of the three algorithms in epoch 100 and use them to generate intrinsic rewards for these two episodes. The variations of intrinsic rewards when picking and stacking objects are shown in Figure 5A. It shows that the intrinsic rewards from three algorithms have a broadly similar trend with slight differences. High intrinsic rewards are generated in special and key operations, e.g., ➀, ➃, and ➆ (gripper closing), ➁ and ➄ (object lifting). However, compared with ICM and RND, which prefer to reward the critical nodes (e.g., ➁, ➄, and ➆), our MIHM tends to reward the whole controllable and important operation processes (e.g., ➀ → ➁ and ➃ → ➄). Moreover, compared with lifting an object, lowering an object is given lower intrinsic rewards (➁ → ➂ and ➄ → ➅). The variations of intrinsic rewards when pushing objects off the table are shown in Figure 5B. Different from ICM and RND that generate high intrinsic rewards when an object falls off the table (➂, ➄, and ➅), our MIHM gives these uncontrollable and dangerous operations low intrinsic rewards. Comparatively, a controllable pull (➃) that prevents the green object from dropping gains higher reward in our MIHM. Figure 5 proves that our MIHM can effectively reward novel behaviors and prevent uncontrollable operations, successfully solving the defect of uncontrollability in the previous curiosity-motivated methods.

**Figure 6:**
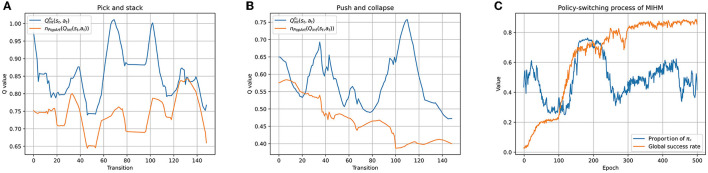
Execution details of hybrid policy mechanism. **(A, B)** Show comparisons between two normalization approaches for constructing the combined Q-function. Compared with the outputs of $Q_{int}^{n_{r}}\left(s_{t},a_{t}\right)$, the outputs of *n*_*PopArt*_(*Q*_*int*_(*s*_*t*_, *a*_*t*_)) are smoother and closer to zero. **(C)** Shows the policy-switching process when training the RL agent by MIHM. The proportion of π_*d*_ fluctuates around 0.5 and the agent can dynamically switch between exploration and exploitation as needed.

In the hybrid policy mechanism of our MIHM, to construct the combined Q-function *Q*_*c*_(*s*_*t*_, *a*_*t*_, *g*), we propose adopting the PopArt-normalized Q-function *n*_*PopArt*_(*Q*_*int*_(*s*_*t*_, *a*_*t*_)) to replace the reward-normalized $Q_{int}^{n_{r}}\left(s_{t},a_{t}\right)$. To show the effect of our proposal, we maintain the two types of Q-functions in the training process and store them in epoch 100. We record their Q-value outputs for the above two artificially controlled episodes in Figures 6A, B. It is shown that the two curves have similar trends that are broadly consistent with the trends of intrinsic rewards in Figures 5A, B, which proves that both Q-functions can effectively learn from intrinsic rewards. However, compared with the outputs of $Q_{int}^{n_{r}}\left(s_{t},a_{t}\right)$, the outputs of *n*_*PopArt*_(*Q*_*int*_(*s*_*t*_, *a*_*t*_)) are smoother and closer to zero, which are more beneficial to the optimization of the DNN-based networks. Based on the PopArt-normalized hybrid reward architecture, when training the RL agent, we record the policy-switching process between the goal-pursuing exploitation policy π_*d*_ and the combined exploration policy π_*c*_. Figure 6C shows the epoch-averaged duration proportion of π_*d*_ in the training rollouts. Because *n*_*PopArt*_(*Q*_*int*_(*s*_*t*_, *a*_*t*_)) is normalized and fluctuates around zero from a macro perspective, the proportion of π_*d*_ fluctuates around 0.5. An interesting point we find is that a rapid rise of the success rate curve often corresponds to more utilization of the exploitation policy π_*d*_ (epoch 0 to 40, epoch 100 to 200), because at that time the agent finds skills for some goals and tends to consolidate them. When the growth of success rate slows down, the agent turns to make more use of the exploration policy π_*c*_ (epoch 40 to 100, epoch 200 to 300). The above phenomena prove that our MIHM can dynamically switch between exploration and exploitation as needed, which is helpful to solve the defect of distraction in the previous curiosity-motivated methods.

**Figure 7:**
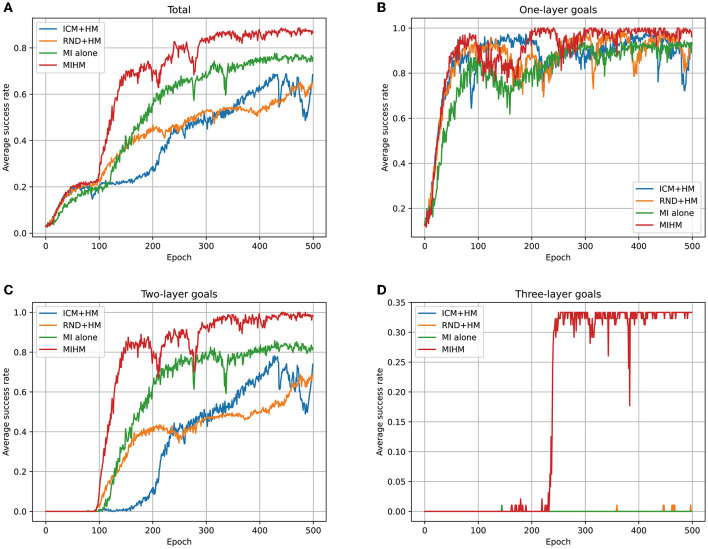
Learning processes of ICM+HM, RND+HM, MI alone and MIHM for different categories of goals. **(A)** Shows the variations of average success rates of all 35 goals. **(B–D)** Show the variations of the average success rates of one-layer goals, two-layer goals and three-layer goals, respectively. Compared with original ICM and RND, ICM+HM and RND+HM increase the final success rates of one-layer goals and two-layer goals by approximately 10% and 30%; MI alone increases the final success rates of one-layer goals and two-layer goals by 10% and 50%. These results prove that overcoming either uncontrollability or distraction can improve the performance of curiosity-motivated methods.

Furthermore, we perform ablation experiments to test the respective performance of the two components of our MIHM: mutual information motivation (MI) and hybrid policy mechanism (HM). Based on the existing ICM, RND and our MIHM, we perform three additional algorithms: ICM+HM, RND+HM and MI alone. The learning processes of different goal categories are recorded in Figure 7. The number of learned semantic goals (whose success rates are >80%) for each category is shown in Table 3. Compared with original ICM and RND in Figure 4, taking advantage of HM, ICM+HM and RND+HM learn faster and increase the final success rates of one-layer goals and two-layer goals by ~10 and 30%, which proves overcoming the defect of distraction can effectively improve the performance of previous curiosity-motivated methods. Moreover, although MI alone has performance degradation with respect to MIHM, it still shows better performance than ICM and RND in Figure 4, especially for the two-layer goals (a 50% increasement in the final success rate), which proves that uncontrollability is a critical obstacle for previous curiosity-motivated methods to dealing with hard manipulation tasks. Compared with ICM + HM and RND + HM, MI alone still has advantage in the final success rate, but it learns slower than RND+HM in the early stage. We think this is because MI alone considers the controllability of the action, which makes its exploration more conservative than RND. In addition, none of the three additional algorithms can achieve the three-layer goals. The combination of MI and HM is necessary for these very hard goals.

**Table 3:** The number of finally learned goals (whose success rates are >80%) in each goal category for ICM+HM, RND+HM, MI alone and MIHM.

**Algorithms**	**One-layer goals**	**Two-layer goals**	**Three-layer goals**	**Total**
ICM+HM	7	6	0	13
RND+HM	8	6	0	14
MI alone	8	17	0	25
MIHM	8	21	2	31

In addition, apart from curiosity-based methods, there exist other possible methods for sparse-reward GCRL. In our robotic manipulation task with semantic goals, we compare the numbers of learned semantic goals of our MIHM with the curriculum learning method DECSTR (Akakzia et al., 2021) and the improved HER method Multi-criteria HER (Lanier et al., 2019). As Table 4 shows, DECSTR achieves 3 more three-layer goals than our MIHM, but its performance is heavily based on task-specific prior knowledge. Multi-critiria HER achieves better performance than vanilla SAC+HER in Table 2, but it still cannot be competent for the semantic-GCRL, though it is designed specifically for the manipulation task. Comparatively, our MIHM does not rely on much task-specific prior knowledge and has few hyperparameters to be determined, which makes it easy to be implemented for more manipulation tasks.

**Table 4:** The number of finally learned goals (whose success rates are >80%) in each goal category for MIHM, DECSTR and Multi-criteria HER.

**Algorithms**	**One-layer goals**	**Two-layer goals**	**Three-layer goals**	**Total**
MIHM	8	21	2	31
DECSTR	8	21	5	34
Multi-criteria HER	3	0	0	3

## 6. Conclusion and future work

Learning semantic-goal-conditioned robotic manipulation with sparse rewards poses a great challenge to the RL training process, because the RL agent will be trapped in the hard exploration problem without sufficient learning signals. In this paper, we leverage the curiosity-motivated methods to intrinsically generate learning signals and facilitate agent exploration. We propose a conservative curiosity-motivated method named mutual information motivation with hybrid policy mechanism (MIHM), which effectively solves the two defects of previous curiosity-motivated methods: uncontrollability and distraction. Different from the previous methods that mainly focus on the generation of intrinsic rewards, we consider improving the entire intrinsically motivated training process, including the quality of the intrinsic rewards and the utilization method of curiosity-motivated exploration. Benefitting from the above improvements, our MIHM shows much better performance than the state-of-the-art curiosity-motivated methods in the semantic-goal-conditioned robotic manipulation task. We believe our method is novel and valuable for all the researchers interested in sparse-reward GCRL.

Nevertheless, there still exists future work for the further improvement of our MIHM. Firstly, in the decoupled-mutual-information-based intrinsic rewards, the forward dynamics prediction model is used to estimate the action uncontrollability. The enhancement of the prediction and generalization capability of this DNN-based model and the acceleration of its convergence rate are beneficial to further reducing the estimation errors from the deficiently trained or incompetent model. Secondly, when training the combined policy π_*c*_, the proportionality coefficient τ for the two Q-functions is static and predefined. We think that if the coefficient can be dynamically adjusted throughout the training process with the avoidance of the possible training instability of π_*c*_, the external rewards and intrinsic rewards will be more sufficiently utilized to improve the global learning efficiency. In general, MIHM in this paper improves some of the components (the generation and exploitation of intrinsic rewards) in the whole RL process, we are interested in combining MIHM with other learning techniques to improve more RL components and better overcome the sparse reward problem.

## Data availability statement

The raw data supporting the conclusions of this article will be made available by the authors, without undue reservation.

## Author contributions

CH contributed to conceptualization, methodology, software, and draft-writing of the study. ZP contributed to validation, formal analysis, and draft-writing of the study. YL contributed to visualization, investigation, draft-editing, and funding acquisition of the study. JT contributed to data curation of the study. YY contributed to project administration of the study. ZZ contributed to supervision of the study. All authors contributed to the article and approved the submitted version.
